# Association between the dietary inflammatory index and pregnancy outcomes in women with gestational diabetes mellitus: a prospective cohort study

**DOI:** 10.3389/fnut.2026.1838318

**Published:** 2026-06-26

**Authors:** Xiaoqian Chen, Wenzhao Lin, Chengyu Huang, Xiaomeng Xue, Yongfan Chen, Yan Bian, Xiumin Jiang

**Affiliations:** 1 Department of Nursing, Fujian Maternity and Child Health Hospital College of Clinical Medicine for Obstetrics and Gynecology and Pediatrics, Fujian Medical University, Fuzhou, China; 2 Department of Nursing, Fujian Obstetrics and Gynecology Hospital, Fuzhou, China; 3 School of Nursing, Fujian Medical University, Fuzhou, China; 4 Labour Ward, Fujian Maternity and Child Health Hospital College of Clinical Medicine for Obstetrics and Gynecology and Pediatrics, Fujian Medical University, Fuzhou, China

**Keywords:** cohort study, dietary inflammatory index, fetal distress, gestational diabetes mellitus, pregnancy outcomes

## Abstract

**Background:**

The study aimed to examine the association between late-pregnancy Dietary Inflammatory Index (DII) and pregnancy outcomes among women with gestational diabetes mellitus (GDM), offering evidence for dietary risk stratification in this high-risk group.

**Methods:**

A prospective cohort study was conducted, enrolling 295 GDM women who received antenatal care in Fujian Province between January and July 2023, with subsequent follow-up until delivery. Dietary intake was assessed using a semi-quantitative food frequency questionnaire to calculate DII, which was grouped into Tertile 1 (T1), Tertile 2 (T2), and Tertile 3 (T3). Pregnancy outcomes and clinical data were obtained from the medical records. Multivariable logistic regression and Firth’s penalized logistic regression were applied to assess associations between DII tertiles and eight pregnancy outcomes, adjusting for maternal age, pre-pregnancy BMI, primiparity, gestational weight gain, and total energy intake. Restricted cubic spline analyses were performed to evaluate potential non-linear relationships.

**Results:**

DII scores ranged from −3.180 to 2.640. Compared to T1, women in T2 had a lower risk of fetal distress in adjusted models [OR = 0.171 (95% CI 0.035–0.848)], while no consistent association was observed for T3. Trend analysis did not indicate a linear relationship between DII and fetal distress. Restricted cubic spline analyses suggested a possible non-linear association. No significant associations were found between DII and other pregnancy outcomes.

**Conclusion:**

In pregnant women with GDM, the intermediate DII group (T2) was associated with a lower risk of fetal distress compared with the lowest DII group (T1), while no significant associations were observed for other pregnancy outcomes. These findings suggest that dietary inflammatory potential may play a potentially complex role in fetal distress among women with GDM, warranting further investigation and consideration in dietary management.

## 1 Introduction

Gestational diabetes mellitus (GDM) is one of the most prevalent metabolic disorders in pregnancy, characterized by glucose intolerance first recognized during gestation (1). A recent systematic review indicated a prevalence of 14.8% for GDM among women in mainland China (2), highlighting its significant public health implications. GDM elevates the risks of cesarean delivery and gestational hypertension, while also demonstrating a strong association with adverse neonatal outcomes such as preterm birth, large-for-gestational-age offspring, and macrosomia (3–5). Furthermore, neonatal respiratory distress syndrome exhibits markedly elevated incidence rates, with reported increases of up to 23.7-fold compared to non-GDM pregnancies (6). These findings emphasize the critical importance of identifying modifiable risk factors to reduce GDM-associated adverse outcomes.

Accumulating evidence indicates that chronic low-grade inflammation plays a central role in the onset and progression of GDM, manifested by exacerbated insulin resistance and elevated pro-inflammatory cytokines (7, 8). Inflammatory dysregulation not only disrupts maternal metabolic homeostasis but may also impair placental function and intrauterine immune balance, thereby influencing fetal growth (9, 10) and contributing to adverse outcomes such as preterm birth (11). As a modifiable and safe external factor, diet has received increasing scientific attention. Epidemiological studies indicated that diet could modulate systemic chronic inflammation through the pro- or anti- inflammatory properties of individual nutrients, specific foods, and overall dietary patterns (12–14).

The Dietary Inflammatory Index (DII), the first standardized framework developed to quantify the pro- and anti-inflammatory properties of diet, provides an innovative metric for assessing the inflammatory potential of an individual’s dietary intake. Its scoring algorithm captures the extent to which dietary exposures influence systemic inflammatory responses (15). DII has been widely applied to investigate associations between diet and risks of cancer (16, 17), diabetes (18, 19), and cardiovascular diseases (20). Emerging studies have linked DII to adverse maternal and neonatal outcomes, while those findings remained inconsistent (21, 22). For instance, Li et al. reported that lower DII scores were associated with a reduced risk of macrosomia (23), whereas Navarro et al. observed no significant association between them (24). Evidence regarding DII specifically in GDM populations remains scarce. Given that women with GDM are simultaneously exposed to heightened inflammation and metabolic dysregulation, the independent contribution of dietary inflammatory potential to pregnancy outcomes in this group warrants further evaluation.

Therefore, this study aimed to investigate the association between DII and pregnancy outcomes among women with GDM. Clarifying the predictive value of DII in this high-risk population may inform targeted dietary intervention strategies and ultimately improve maternal and neonatal health.

## 2 Materials and methods

### 2.1 Study design and participants

Data for this study were obtained from a prospective cohort conducted in Fujian Province between January and July 2023. The primary aim was to evaluate the association between the DII in late pregnancy and pregnancy outcomes among women with GDM. Sample size estimation was based on an overall adverse pregnancy outcome rate of 37.86% reported in Chinese women with GDM (25). A minimum events-per-variable (EPV) criterion of ≥10 was applied as a conservative guideline for multivariable logistic regression models (26). After accounting for an anticipated 10% loss to follow-up, the final sample size (n = 295) was considered adequate to meet the EPV requirement for the planned analyses.

The diagnosis of GDM was made in accordance with the guidelines recommended by the International Association of Diabetes and Pregnancy Study Groups (IADPSG) (27). Inclusion criteria were as follows: ① age ≥18 years; ② late pregnancy; ③ regular attendance at routine antenatal examinations and planned delivery at the study hospital; ④ no communication barriers; and ⑤ absence of severe pregnancy complications or comorbid conditions.

Given the limited number of outcome events for certain endpoints, multivariable analyses were performed using a parsimonious set of *a priori* selected covariates based on clinical relevance and prior literature to minimize overfitting. Outcomes with limited events were interpreted with caution. For the fetal distress outcome, which involved 22 events, only a limited set of prespecified covariates was included in the multivariable models, and Firth’s penalized logistic regression was additionally performed as a sensitivity analysis to reduce potential small-sample bias.

This study was approved by the Ethics Committee of Fujian Provincial Maternity and Child Health Hospital (Approval No. 2023KY051). All participants provided written informed consent, and data collection was conducted in an anonymized manner. The study followed the guidelines set forth in the STROBE (Strengthening the Reporting of Observational Studies in Epidemiology) statement for comprehensive and transparent reporting of observational studies.

### 2.2 Dietary assessment and DII scoring

This study assessed maternal dietary intake using a semi-quantitative food frequency questionnaire (SQ-FFQ). The instrument captured intake over the preceding 28 days, documenting the frequency, types, and quantities of food consumption. The SQ-FFQ was specifically tailored to Chinese dietary habits and nutrient profiles, comprising 61 food items that represent more than 200 food groups, and has demonstrated strong reliability in previous research (28). To minimize recall bias, trained investigators used color food photographs and standardized food models to support accurate quantification of intake. Daily energy and nutrient intakes were subsequently calculated based on the 2009 Chinese Food Composition Table (29).

The resulting dietary nutrient data were used to compute each participant’s DII score. DII values were derived following the scoring algorithm developed by Shivappa et al. (15). For each dietary parameter, a Z-score was first generated by comparing the individual’s intake with the global mean and standard deviation obtained from 11 international populations. To mitigate the influence of right-skewed distributions, each Z-score was converted to a percentile, doubled, and then reduced by one, yielding a centered distribution ranging from −1 to 1. The DII score for each nutrient was obtained by multiplying this centered percentile score by its corresponding inflammatory effect score. The total DII was calculated as the sum of all nutrient-specific DII values. Higher DII scores indicate a more pro-inflammatory dietary profile (15).

The original DII algorithm includes 45 dietary parameters. In accordance with dietary characteristics of Chinese populations, this study quantified the intake of 27 dietary energy and nutrient components, including energy, protein, total fat, carbohydrates, fiber, cholesterol, vitamins A, B_1_, B_2_, B_3_, B_6_, B_12_, C, D, and E, soy isoflavones, saturated fatty acids (SFA), monounsaturated fatty acids (MUFA), polyunsaturated fatty acids (PUFA), iron, zinc, selenium, magnesium, n-3 PUFA, n-6 PUFA, folic acid, and β-carotene. The remaining 18 parameters (e.g., alcohol, caffeine, selected spices, tea, and various polyphenols) were not available in the FFQ and were therefore excluded. Previous evidence indicates that even when fewer than 30 nutrients are included, the predictive validity of the DII remains robust (30). A detailed comparison of all 45 original components and their availability in this study is provided in 1Supplementary Table S1.

For sensitivity analyses, the energy-adjusted dietary inflammatory index (E-DII) was calculated by standardizing dietary intakes per 1,000 kcal before applying the same DII algorithm, allowing assessment of dietary inflammatory potential independent of total energy intake. The FFQ was administered in late pregnancy (gestational age ≥28 weeks), capturing habitual intake over the previous 28 days. Dietary data were obtained through one-to-one face-to-face interviews with real-time completion of missing or inconsistent responses, resulting in complete dietary exposure data.

### 2.3 Exposure

Participants with GDM were stratified into three groups according to the tertile distribution of total DII scores: the lowest tertile (Tertile 1, T1), the middle tertile (Tertile 2, T2), and the highest tertile (Tertile 3, T3). This tertile-based categorization was performed with reference to previous studies on the DII (31, 32) and was applied to facilitate comparisons across increasing levels of dietary inflammatory potential and to reduce the influence of extreme values. Demographic characteristics and pregnancy outcomes were subsequently compared across these tertiles (T1–T3). The specific cut-off values for DII and E-DII tertiles are presented in Table 1.

**Table 1:** General characteristics of GDM pregnant women in different DII groups (*n* = 295).

Characteristics	All (n = 295)	DII			*F/χ^2^*	*P*
		Tertile 1 (*n* = 100)	Tertile 2 (*n* = 97)	Tertile 3 (*n* = 98)		
DII	0.068 ± 1.444	−1.515 ± 0.531	−0.008 ± 0.529	1.744 ± 0.576	882.758	**<0.001**
DII range	−3.180 ~ 2.640	−3.180 ~ −0.850	−0.840 ~ 0.870	0.900 ~ 2.640	/	/
Age (y)					8.954	0.175
<25	9 (3.1)	2 (2.0)	2 (2.0)	5 (5.1)		
25–29	72 (24.4)	19 (19.0)	23 (23.7)	30 (30.6)		
30–34	140 (47.5)	57 (57.0)	44 (45.4)	39 (39.8)		
≥35	74 (25.1)	22 (22.0)	28 (28.9)	24 (24.5)		
Educational level					6.739	0.349
Junior high school and below	22(7.5)	8(8.0)	6(6.2)	8(8.2)		
High school or vocational school	59(20.0)	21(21.0)	14(14.4)	24(24.5)		
Junior college	73(24.7)	22(22.0)	23(23.7)	28(28.6)		
Bachelor’s degree and above	141(47.8)	49(49.0)	54(55.7)	38(38.8)		
Average monthly personal income (CNY)					11.014	0.201
<3,000	5(1.7)	2(2.0)	1(1.1)	2(2.0)		
3,000~5,999	65(22.0)	15(15.0)	23(23.7)	27(27.6)		
6,000~8,999	102(34.6)	34(34.0)	29(29.9)	39(39.8)		
9,000~11,999	66(22.4)	28(28.0)	21(21.6)	17(17.3)		
≥12,000	57(19.3)	21(21.0)	23(23.7)	13(13.3)		
Occupation					11.232	0.668
Unemployed	72(24.4)	27(27.0)	17(17.5)	28(28.6)		
Office worker	91(30.8)	26(26.0)	33(34.0)	32(32.7)		
Civil servant	10(3.4)	3(3.0)	5(5.2)	2(2.0)		
Service industry	21(7.1)	8(8.0)	7(7.2)	6(6.1)		
Medical personnel	6(2.0)	2(2.0)	2(2.1)	2(2.0)		
Self-employed	15(5.1)	3(3.0)	8(8.2)	4(4.1)		
Teacher	34(11.5)	11(11.0)	15(15.5)	8(8.2)		
Other	46(15.6)	19(19.0)	11(11.3)	16(16.3)		
Nutritional counseling frequency					13.517	**0.009**
Never	128(43.4)	34(34.0)	40(41.3)	54(55.1)		
1–2 times	129(43.7)	56(56.0)	43(44.3)	30(30.6)		
≥3 times	38(12.9)	10(10.0)	14(14.4)	14(14.3)		
Pre-pregnancy BMI (kg/m^2^)					5.441	0.495
Underweight (<18.5)	15(5.1)	6(6.0)	2(2.1)	7(7.1)		
Normal weight (18.5~)	184(62.4)	65(65.0)	59(60.8)	60(61.2)		
Overweight (24~)	65(22.0)	18(18.0)	27(27.8)	20(20.4)		
Obese (≥28)	31(10.5)	11(11.0)	9(9.3)	11(11.2)		
OGTT blood glucose (mmol/L)						
OGTT-0 h	4.94 ± 0.52	4.98 ± 0.59	4.98 ± 0.55	4.85 ± 0.40	1.811	0.165
OGTT-1 h	10.30 ± 1.55	10.42 ± 1.45	10.52 ± 1.61	9.95 ± 1.54	3.861	**0.022**
OGTT-2 h	8.80 ± 1.58	9.06 ± 1.72	8.87 ± 1.53	8.45 ± 1.41	4.043	**0.019**
HbA1c	5.36 ± 0.34	5.40 ± 0.33	5.33 ± 0.36	5.34 ± 0.31	1.151	0.318
Primiparity status					3.802	0.148
No	114(38.6)	41(41.0)	30(30.9)	43(43.9)		
Yes	181(61.4)	59(59.0)	67(69.1)	55(56.1)		
Adverse pregnancy history					2.849	0.245
No	200(67.8)	62(62.0)	71(73.52)	67(68.4)		
Yes	95(32.2)	38(38.0)	26(26.8)	31(31.6)		
Smoking history					/	0.848^*^
No	291(98.6)	99(99.0)	96(99.0)	96(98.0)		
Yes	4(1.4)	1(1.0)	1(1.0)	2(2.0)		
Drinking history					/	1.000^*^
No	290(98.3)	98(98.0)	96(99.0)	96(98.0)		
Yes	5(1.7)	2(2.0)	1(1.0)	2(2.0)		

DII, Dietary Inflammatory Index; CNY, Chinese Yuan; BMI, body mass index; GDM, gestational diabetes mellitus; OGTT, oral glucose tolerance test; SD, standard deviation. Continuous variables are expressed as mean ±standard deviation, and categorical variables are expressed as n (%). F: the statistics of one-way analysis of variance. χ2: the statistics of the chi-square test. * Fisher’s exact test. Bold values indicate that the *p*-value is less than 0.05, indicating statistical significance. Participants were categorized into tertiles based on DII distribution. Cut-off values were as follows: DII (T1 ≤ −0.850, T2 –0.850 to 0.870, T3 > 0.870).

### 2.4 Pregnancy outcomes

Pregnancy outcomes assessed in this study included low birth weight, macrosomia, appropriate for gestational age, small for gestational age, fetal distress, preterm delivery, premature rupture of membranes, and cesarean section. Standardized definitions were adopted based on established literature:

① Low birth weight (LBW): neonatal weight <2,500 g measured within 1 h after delivery; ② Macrosomia: neonatal weight ≥4,000 g measured within 1 h after delivery (33); ③ Appropriate for gestational age (AGA): birth weight between the 10th and 90th percentiles for infants of the same sex and gestational age (34); ④ Small for gestational age (SGA): birth weight below the 10th percentile for infants of the same sex and gestational age (34); ⑤ Fetal distress: fetal distress was identified according to the diagnosis recorded in the electronic medical records by the attending obstetrician. In routine clinical practice at the study hospital, the diagnosis is primarily based on abnormal fetal heart rate patterns suggestive of fetal hypoxia (e.g., late decelerations, fetal tachycardia, or variable decelerations), together with overall clinical assessment. ⑥ Preterm delivery: delivery occurring between 28 and <37 completed gestational weeks (35); ⑦ Premature rupture of membranes (PROM): rupture of membranes before the onset of regular uterine contractions (36); ⑧ Cesarean section: delivery of the fetus through incisions made in the abdominal and uterine walls.

### 2.5 Assessment of covariates

At enrollment, trained researchers systematically collected potential confounding variables using a structured questionnaire and the hospital’s electronic medical record system.

Continuous variables included pre-pregnancy body mass index (BMI), gestational weight gain, total energy intake, OGTT blood glucose, and glycated hemoglobin (HbA1c). Age was categorized as <25, 25–29, 30–34, and ≥35 years based on clinical relevance. Educational level was classified as junior high school and below, high school or vocational school, junior college and bachelor’s degree and above. Average monthly personal income was grouped as <3,000; 3,000–5,999; 6,000–8,999; 9,000–11,999; and ≥12,000 RMB. Occupations were categorized as unemployed, office worker, civil servant, service industry, medical personnel, self-employed, teacher and other. Nutritional counseling frequency was classified as never, 1–2 times, and ≥3 times. Pre-pregnancy BMI was categorized as underweight, normal weight, overweight, or obese according to the Chinese Working Group on Obesity guidelines (37). Smoking, alcohol consumption, primiparity, and history of adverse pregnancy outcomes were treated as binary variables. Adverse pregnancy history referred to a history of miscarriage, fetal malformation, or stillbirth.

To reduce the risk of overfitting and improve model stability, particularly for outcomes with limited events, multivariable models were adjusted for a small set of key confounders selected *a priori* based on clinical relevance and prior literature (38, 39). Maternal age, pre-pregnancy BMI, primiparity status, and gestational weight gain were included as core confounders. Total energy intake was additionally included in an extended model.

Other collected variables were used for descriptive analyses but were not included in primary models to avoid overadjustment. Sensitivity analyses further adjusted for nutritional counseling frequency and OGTT glucose levels (1-h and 2-h), as these variables differed across DII tertiles and may reflect dietary behavior modification and glycemic status during pregnancy. For outcomes with relatively few events, such as fetal distress, Firth’s penalized logistic regression was further applied to improve estimate stability.

### 2.6 Data collection

During recruitment, trained researchers explained the study’s objectives, significance, and procedures to participants with gestational diabetes mellitus. After obtaining informed consent, sociodemographic characteristics, lifestyle behaviors, and dietary intake were collected using a structured questionnaire. Clinical information, including pre-pregnancy weight, admission weight for delivery, gestational weight gain, oral glucose tolerance test (OGTT) results, glycated hemoglobin (HbA1c), and obstetric history, was extracted from the electronic medical record system to ensure data accuracy and completeness. Dietary intake during pregnancy was assessed using a validated semi-quantitative food frequency questionnaire. Trained interviewers employed standardized food models and color photographic aids to enhance portion-size estimation and minimize recall bias and interviewer variability.

### 2.7 Statistical analysis

Participants with missing outcome data were excluded. No imputation was performed, and a complete-case analysis was applied. All statistical analyses were conducted using SPSS version 27.0 and R software (version 4.5.3). Continuous variables with a normal distribution were expressed as mean ± standard deviation (SD), whereas non-normally distributed variables were presented as median with interquartile range [M(P_25_, P_75_)]. Categorical variables were summarized as counts and percentages [n(%)]. Between-group comparisons for normally distributed continuous variables were conducted using independent t-tests or analysis of variance (ANOVA), while categorical variables were compared using the chi-square test or Fisher’s exact test as appropriate.

Logistic regression analyses were conducted to evaluate the associations between DII and pregnancy outcomes. We assessed potential departures from linearity by modeling DII as both a continuous variable and categorical tertiles, and further explored non-linear associations using restricted cubic spline models. Pregnancy outcomes among women with GDM served as dependent variables, with DII tertiles (reference group: T1) and total DII scores as independent variables. Age, pre-pregnancy BMI, primiparity status, and gestational weight gain were selected *a priori* as key confounders based on prior literature and clinical relevance and were included in the primary multivariable models, with total energy intake additionally adjusted for in extended models.

For outcomes with relatively low event numbers, Firth’s penalized logistic regression was applied to reduce potential small-sample bias. Influence diagnostics, including Cook’s distance and leverage statistics, were additionally performed based on the fully adjusted logistic regression model of fetal distress to evaluate the potential impact of individual observations. Sensitivity analyses were conducted using the energy-adjusted dietary inflammatory index (E-DII), with similar modeling strategies applied. Trend analyses were conducted to assess dose–response relationships between DII tertiles and pregnancy outcomes. For trend analysis, the median DII value of each tertile was treated as a continuous variable in logistic regression models, and *P _for trend_* values were calculated to assess trends across categories. RCS analyses were performed for fetal distress to explore potential non-linear associations between dietary inflammatory indices and fetal distress. Four knots were placed at the 5th, 35th, 65th, and 95th percentiles of the DII and E-DII distributions. Odds ratios (ORs) with 95% confidence intervals (CIs) were estimated using the median value of each exposure distribution as the reference. Wald *χ^2^* tests were used to assess overall and non-linear associations. All tests were two-sided, and statistical significance was defined as *p* ≤ 0.05.

## 3 Results

**Figure 1:**
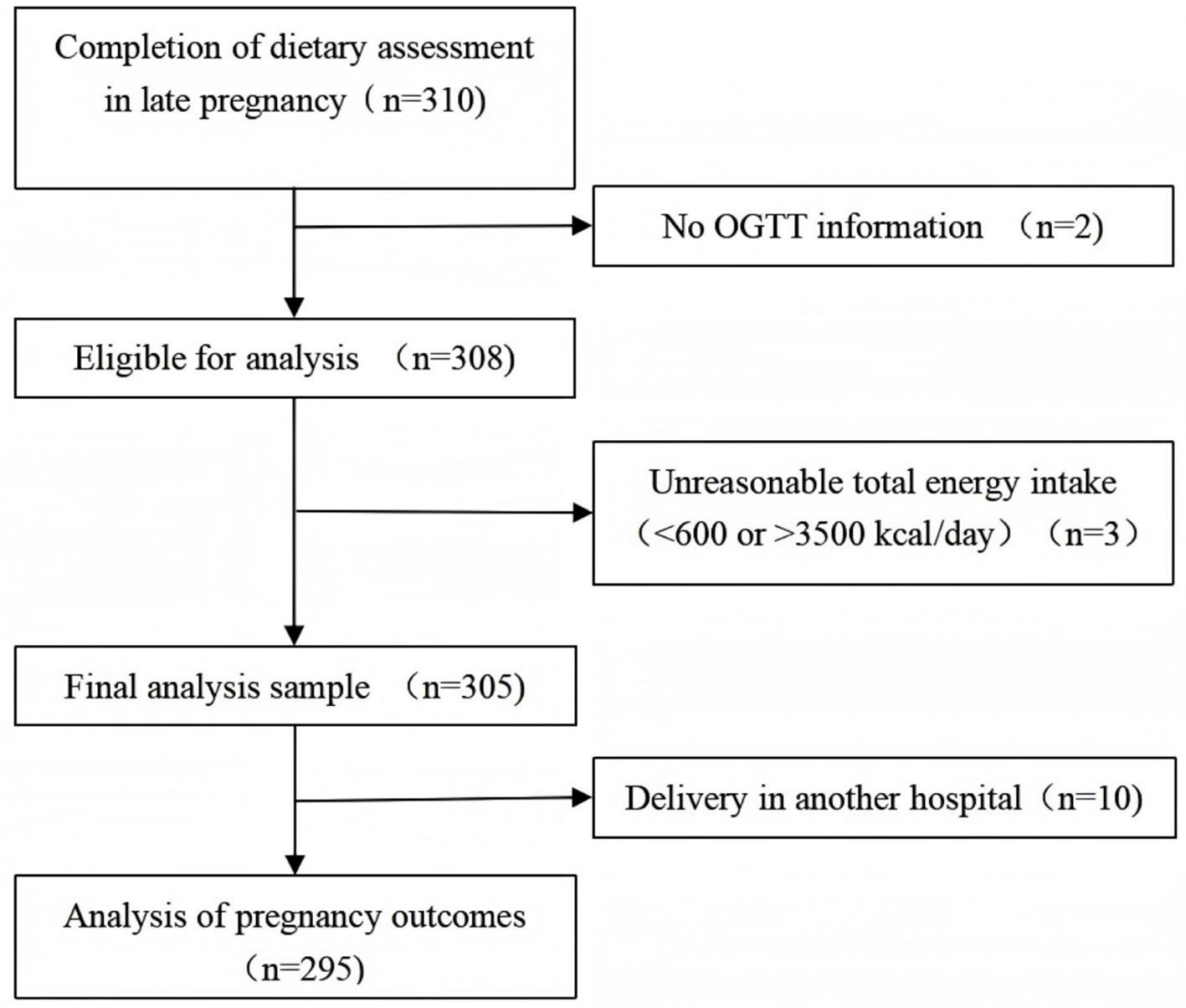
Flowchart of participant inclusion in the study.

A total of 310 women in late pregnancy were initially recruited. Two participants were excluded due to missing OGTT data, and three were excluded for abnormal dietary energy intake (<600 kcal/day or >3,500 kcal/day). Ultimately, 305 participants completed the baseline survey, of whom 10 delivered at external hospitals and were excluded due to unavailable pregnancy outcome data. Therefore, 295 participants were included in the final analysis of pregnancy outcomes. The detailed participant inclusion flowchart is presented in Figure 1.

### 3.1 General characteristics of pregnant women with GDM

The overall mean DII among women with GDM was 0.068 ± 1.444, ranging from −3.180 to 2.640. Maternal age was predominantly 30–34 years (47.5%), 47.8% had a bachelor’s degree or higher, and 61.4% were primiparous. The gestational age at the time of dietary assessment was 30.31 ± 2.66 weeks. Comparison of demographic and clinical variables across DII tertiles revealed statistically significant differences in nutritional counseling frequency and glucose levels at the 1-h and 2-h OGTT time points (*p* < 0.05). No statistically significant differences were observed among tertiles in age, educational level, average monthly personal income, occupation, primiparity status, or other lifestyle-related factors (all *p* > 0.05). Detailed results are provided in Table 1.

### 3.2 Nutrient intake among GDM pregnant women stratified by DII scores

The distribution of dietary nutrient intake across different DII score groups is presented in Table 2. Significant differences (*p* < 0.05) were observed among the three groups for the intake of energy, protein, total fat, carbohydrate, fiber, cholesterol, vitamins A, B_1_, B_2_, B_3_, B_6_, B_12_, D, and E, isoflavones, SFA, MUFA, PUFA, iron, zinc, selenium, magnesium, n-3 PUFA, n-6 PUFA, folic acid, and *β*-carotene.

**Table 2:** Nutrient intake of pregnant women with GDM in different DII score groups (*n* = 295).

Dietary parameters	DII ( $\bar{x}\pm s$ )				*F*	*P*
	All (*n* = 295)	Tertile 1 (*n* = 100)	Tertile 2 (*n* = 97)	Tertile 3 (*n* = 98)		
Energy(kcal/d)	1391.57 ± 326.27	1578.39 ± 322.96	1361.56 ± 287.87	1230.66 ± 266.73	35.450	<0.001
Protein (g/d)	68.32 ± 19.05	82.53 ± 19.32	66.76 ± 15.37	55.37 ± 10.25	77.088	<0.001
Total fat(g/d)	44.91 ± 18.38	50.63 ± 17.64	46.52 ± 17.86	37.48 ± 17.27	14.437	<0.001
Carbohydrate (g/d)	206.63 ± 60.41	227.99 ± 63.73	201.62 ± 51.04	189.78 ± 59.61	11.116	<0.001
Fiber (g/d)	8.76 ± 3.23	11.18 ± 3.66	8.29 ± 2.06	6.76 ± 1.85	70.711	<0.001
Cholesterol (mg/d)	467.93 ± 125.98	513.72 ± 109.13	477.98 ± 127.92	411.25 ± 119.35	18.879	<0.001
Vitamin A (RE/d)	558.22 ± 235.84	749.16 ± 223.46	519.90 ± 141.82	401.30 ± 183.78	89.204	<0.001
Thiamin (mg/d)	1.18 ± 0.28	1.37 ± 0.25	1.16 ± 0.22	1.02 ± 0.23	54.255	<0.001
Riboflavin (mg/d)	1.18 ± 0.29	1.39 ± 0.23	1.15 ± 0.26	0.99 ± 0.22	69.187	<0.001
Niacin (mg/d)	9.92 ± 3.20	12.05 ± 2.98	9.81 ± 2.95	7.84 ± 2.05	60.380	<0.001
Vitamin B_6_ (mg/d)	1.80 ± 0.42	2.15 ± 0.41	1.74 ± 0.30	1.49 ± 0.24	103.498	<0.001
Vitamin B_12_ (μg/d)	4.24 ± 1.36	5.05 ± 1.27	4.04 ± 1.20	3.59 ± 1.20	37.327	<0.001
Vitamin C (μg/d)	107.29 ± 120.06	118.86 ± 49.32	106.95 ± 107.43	95.83 ± 171.79	0.911	0.403
Vitamin D (μg/d)	26.87 ± 11.28	29.65 ± 10.18	26.67 ± 11.31	24.23 ± 11.74	5.940	0.003
Vitamin E (mg/d)	11.56 ± 4.95	15.11 ± 5.92	11.28 ± 2.96	8.22 ± 2.37	71.030	<0.001
Isoflavones (mg/d)	6.38 ± 7.88	9.89 ± 10.60	6.36 ± 5.60	3.03 ± 4.47	19.943	<0.001
Mg (mg/d)	339.27 ± 95.64	429.74 ± 86.90	324.01 ± 50.40	262.05 ± 53.00	164.667	<0.001
Se (μg/d)	42.08 ± 14.26	51.86 ± 16.56	40.09 ± 10.09	34.06 ± 8.24	54.563	<0.001
Fe (mg/d)	12.90 ± 3.89	16.74 ± 3.50	12.29 ± 2.12	9.60 ± 1.53	201.764	<0.001
Zn (mg/d)	9.67 ± 2.77	11.98 ± 2.53	9.35 ± 2.18	7.63 ± 1.50	106.310	<0.001
SFA (g/d)	15.60 ± 4.32	17.79 ± 3.83	15.32 ± 4.24	13.65 ± 3.86	27.219	<0.001
MUFA (g/d)	11.06 ± 3.60	12.89 ± 3.57	10.82 ± 3.38	9.42 ± 2.97	27.356	<0.001
PUFA (g/d)	4.28 ± 2.07	4.78 ± 1.96	4.51 ± 2.16	3.54 ± 1.90	10.253	<0.001
n-3(PUFA) (g/d)	0.56 ± 0.32	0.64 ± 0.33	0.58 ± 0.30	0.46 ± 0.32	9.008	<0.001
n-6(PUFA) (g/d)	3.54 ± 1.65	4.02 ± 1.63	3.69 ± 1.57	2.92 ± 1.56	12.372	<0.001
Folic acid (μg/d)	441.60 ± 169.78	612.50 ± 148.94	407.40 ± 91.47	301.07 ± 72.22	206.995	<0.001
*β*-Carotene (μg/d)	4039.09 ± 2060.65	5919.25 ± 1866.52	3678.74 ± 1358.78	2477.23 ± 1117.35	137.354	<0.001

DII, Dietary Inflammatory Index; MUFA, monounsaturated fatty acids; PUFA, polyunsaturated fatty acids; SFA, saturated fatty acids. Continuous variables are expressed as mean ±standard deviation. F: the statistics of one-way analysis of variance.

### 3.3 Pregnancy outcomes among women with GDM across different DII categories

Based on DII tertiles, 295 women with GDM were stratified into T1, T2, and T3 groups. Comparison of pregnancy outcomes across these categories showed that the incidence of fetal distress was 10.0, 2.1, and 10.2% in T1, T2, and T3, respectively, with a statistically significant difference (*p* = 0.050). No statistically significant differences were observed among the three groups for cesarean delivery, PROM, preterm birth, AGA, SGA, macrosomia, or low birth weight (*p* > 0.05), as shown in Table 3. Given that eight pregnancy outcomes were assessed without adjustment for multiple comparisons, these findings should be considered exploratory and interpreted with caution.

**Table 3:** Pregnancy outcomes in women with GDM according to different DII groups (*n* = 295).

Pregnancy outcomes	All (*n* = 295)	DII			*F/χ^2^*	*P*
		Tertile 1 (*n* = 100)	Tertile 2 (*n* = 97)	Tertile 3 (*n* = 98)		
Gestational weight gain (kg)	10.75 ± 5.00	10.21 ± 4.92	10.45 ± 4.65	11.61 ± 5.35	2.211	0.111
Gestational age (wk)	38.49 ± 1.41	38.40 ± 1.37	38.57 ± 1.25	38.51 ± 1.60	0.356	0.701
Newborn birth weight (g)	3255.39 ± 448.64	3247.40 ± 490.75	3265.26 ± 437.39	3253.78 ± 418.05	0.040	0.961
Cesarean section					0.085	0.967
No	157 (53.2)	54 (54.0)	52 (53.6)	51 (52.0)		
Yes	138 (46.8)	46 (46.0)	45 (46.4)	47 (48.0)		
PROM					0.998	0.609
No	226 (76.6)	79 (79.0)	71 (73.2)	76 (77.6)		
Yes	69 (23.4)	21 (21.0)	26 (26.8)	22 (22.4)		
Preterm delivery					/	0.716^*^
No	283 (95.9)	97 (97.0)	92 (94.8)	94 (95.9)		
Yes	12 (4.1)	3 (3.0)	5 (5.2)	4 (4.1)		
SGA					/	0.724^*^
No	285 (96.6)	96 (96.0)	93 (95.9)	96 (98.0)		
Yes	10 (3.4)	4 (4.0)	4 (4.1)	2 (2.0)		
AGA					/	0.515^*^
No	11 (3.7)	4 (4.0)	5 (5.2)	2 (2.0)		
Yes	284 (96.3)	96 (96.0)	92 (94.8)	96 (98.0)		
Macrosomia					/	0.724^*^
No	285 (96.6)	96 (96.0)	93 (95.9)	96 (98.0)		
Yes	10 (3.4)	4 (4.0)	4 (4.1)	2 (2.0)		
LBW					/	0.456^*^
No	281 (95.3)	93 (93.0)	93 (95.9)	95 (96.9)		
Yes	14 (4.7)	7 (7.0)	4 (4.1)	3 (3.1)		
Fetal distress					6.100	**0.050**
No	273 (92.5)	90 (90.0)	95 (97.9)	88 (89.8)		
Yes	22 (7.5)	10 (10.0)	2 (2.1)	10 (10.2)		

PROM, Premature rupture of membranes; SGA, Small for gestational age; AGA, Appropriate for gestational age; LBW, Low birth weight. Continuous variables are expressed as mean ±standard deviation, and categorical variables are expressed as n (%). F: the statistics of one-way analysis of variance. χ2: the statistics of the chi-square test. * Fisher’s exact test. Bold values indicate that the *p*-value is less than 0.05, indicating statistical significance.

### 3.4 Logistic regression analysis of GDM pregnant women’s DII and pregnancy outcomes

Binary logistic regression analyses were performed to examine the association between the DII and pregnancy outcomes among women with GDM. DII was included in the analyses as a continuous variable and, in separate models, categorized into tertiles (T1–T3), using T1 as the reference group. Three models were constructed: an unadjusted model; Model 1 adjusted for age, pre-pregnancy BMI, primiparity status, and gestational weight gain; and Model 2 additionally adjusted for total energy intake.

In the unadjusted model, women in the T2 group showed a significantly lower risk of fetal distress compared with the T1 group (OR = 0.189, 95% CI: 0.040–0.889, *p* = 0.035). This association remained significant after adjustment, with the risk further attenuated in Model 1 (OR = 0.153, 95% CI: 0.032–0.737, *p* = 0.019) and Model 2 (OR = 0.171, 95% CI: 0.035–0.848, *p* = 0.031). By contrast, neither continuous DII scores nor tertile classifications were associated with the risks of AGA, SGA, macrosomia, low birth weight, preterm birth, cesarean section, or premature rupture of membranes in any model (all *p* > 0.05).

**Figure 2:**
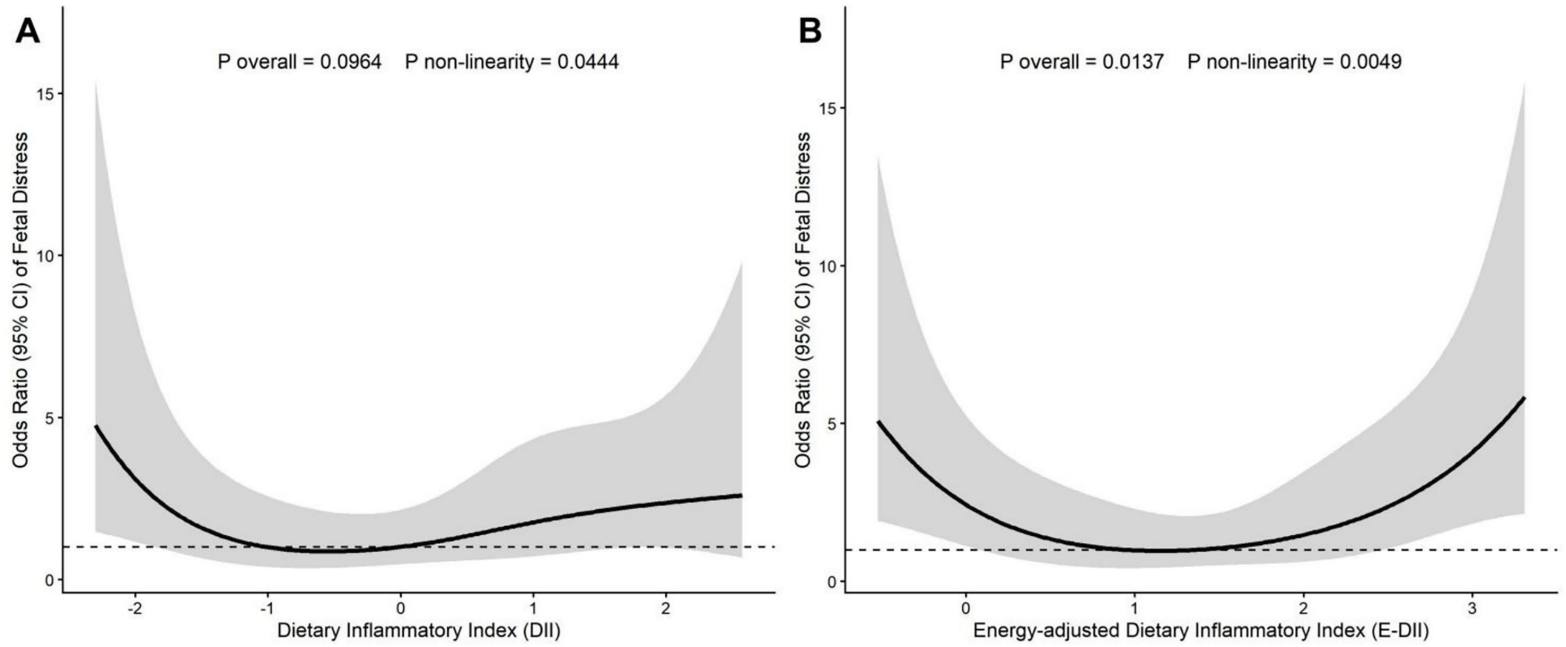
Multivariable dose–response association between dietary inflammatory index (DII) and energy-adjusted dietary inflammatory index (E-DII) with fetal distress among women with gestational diabetes mellitus (GDM). Odds ratios (ORs) with 95% confidence intervals (CIs) were estimated using restricted cubic spline models based on multivariable logistic regression. Models were adjusted for maternal age (continuous, years), pre-pregnancy body mass index (continuous, kg/m^2^), parity (primiparous or multiparous), and gestational weight gain (continuous, kg). Total energy intake (continuous, kcal/day) was additionally adjusted in the DII model. DII and E-DII were modeled as continuous exposures, with the reference value set at the median of each exposure distribution. Solid lines represent ORs, and shaded areas indicate 95% CIs. Four knots were placed at the 5th, 35th, 65th, and 95th percentiles of the DII and E-DII distributions. Panel **(A)** shows the association between DII and fetal distress. Panel **(B)** shows the association between E-DII and fetal distress.

Trend analysis showed no significant linear association between DII scores and any of the eight pregnancy outcomes across all models (all *P _for trend_* > 0.05), supporting the absence of a consistent dose–response relationship. Detailed results are presented in Table 4. The discrepancy between categorical and continuous analyses suggests a potential non-linear association rather than a monotonic linear relationship. Restricted cubic spline (RCS) analyses were further conducted to explore potential non-linear associations between dietary inflammatory indices and fetal distress. For DII, the overall association was not statistically significant (*P _overall_* = 0.0964), whereas a significant non-linear component was detected (*P _for non-linearity_* = 0.0444) (Figure 2A). For E-DII, both the overall association (*P _overall_* = 0.0137) and the non-linear component (*P _for non-linearity_* = 0.0049) were statistically significant (Figure 2B). The spline curves suggested a non-linear dose–response relationship across the observed exposure range, although no prespecified threshold or cut-off value was defined in the model. The apparent inflection points (DII = -0.56 and E-DII = 1.15) should be interpreted with caution, as they were not derived from a segmented regression model or formally estimated change-point analysis, but rather visually inferred from the spline curves, and may be influenced by sparse observations at the distribution tails and the relatively limited number of fetal distress events. This non-linear pattern may account for the significant association observed only in the intermediate tertile (T2), in the absence of a linear trend or continuous association, suggesting a potential non-linear relationship between dietary inflammatory index and fetal distress.

**Table 4:** Logistic regression analysis of DII scores in GDM pregnant women and the risk of pregnancy outcomes.

DII tertiles	Crude model		Adjusted model 1		Adjusted model 2	
	OR (95% CI)	*P*	OR (95% CI)	*P*	OR (95% CI)	P
SGA						
DII (continuous)	0.856 (0.548, 1.339)	0.496	0.933 (0.580, 1.502)	0.776	0.790 (0.438, 1.423)	0.434
Tertile 1	Ref.	-	Ref.	-	Ref.	-
Tertile 2	1.032 (0.251, 4.249)	0.965	1.111 (0.255, 4.845)	0.912	0.871 (0.184, 4.122)	0.862
Tertile 3	0.500 (0.089, 2.794)	0.430	0.644 (0.110, 3.768)	0.716	0.469 (0.063, 3.490)	0.460
*P_for trend_*		0.439		0.748		0.469
AGA						
DII(continuous)	1.164 (0.760, 1.782)	0.486	1.075 (0.684, 1.691)	0.754	1.277 (0.728, 2.237)	0.394
Tertile 1	Ref.	-	Ref.	-	Ref.	-
Tertile 2	0.767 (0.200, 2.944)	0.699	0.736 (0.181, 2.992)	0.669	0.918 (0.210, 4.008)	0.910
Tertile 3	2.000 (0.358, 11.178)	0.430	1.461 (0.244, 8.747)	0.678	2.241 (0.307, 16.342)	0.426
*P_for trend_*		0.450		0.745		0.449
Macrosomia						
DII (continuous)	0.926 (0.596, 1.439)	0.733	0.933 (0.589, 1.479)	0.769	1.000 (0.589, 1.697)	1.000
Tertile 1	Ref.	-	Ref.	-	Ref.	-
Tertile 2	1.032 (0.251, 4.249)	0.965	1.012 (0.230, 4.447)	0.988	1.095 (0.229, 5.224)	0.910
Tertile 3	0.500 (0.089, 2.794)	0.430	0.495 (0.082, 2.970)	0.442	0.564 (0.079, 4.023)	0.568
*P_for trend_*		0.439		0.452		0.579
LBW						
DII (continuous)	0.799 (0.543, 1.177)	0.256	0.855 (0.567, 1.290)	0.456	0.783 (0.475, 1.291)	0.338
Tertile 1	Ref.	-	Ref.	-	Ref.	-
Tertile 2	0.571 (0.162, 2.018)	0.385	0.583 (0.154, 2.201)	0.426	0.515 (0.128, 2.075)	0.350
Tertile 3	0.420 (0.105, 1.672)	0.218	0.585 (0.137, 2.486)	0.467	0.470 (0.093, 2.379)	0.361
*P_for trend_*		0.204		0.417		0.330
Preterm delivery						
DII (continuous)	1.087 (0.728, 1.621)	0.684	1.174 (0.766, 1.799)	0.463	1.114 (0.672, 1.846)	0.675
Tertile 1	Ref.	-	Ref.	-	Ref.	-
Tertile 2	1.757(0.408, 7.563)	0.449	1.615(0.360, 7.252)	0.532	1.473(0.309, 7.027)	0.627
Tertile 3	1.376(0.300, 6.313)	0.681	1.774(0.369, 8.533)	0.475	1.499(0.260, 8.649)	0.651
*P_for trend_*		0.719		0.476		0.665
Cesarean section						
DII (continuous)	0.991 (0.846, 1.162)	0.912	1.009 (0.852, 1.194)	0.919	1.078 (0.886, 1.311)	0.453
Tertile 1	Ref.	-	Ref.	-	Ref.	-
Tertile 2	1.016 (0.580, 1.779)	0.956	0.928 (0.515, 1.674)	0.804	1.059 (0.571, 1.965)	0.855
Tertile 3	1.082 (0.619, 1.891)	0.782	1.190 (0.658, 2.152)	0.565	1.473 (0.759, 2.860)	0.252
*P_for trend_*		0.780		0.561		0.243
Fetal distress						
DII (continuous)	0.928 (0.685, 1.257)	0.630	0.920 (0.669, 1.265)	0.608	0.963 (0.663, 1.398)	0.843
Tertile 1	Ref.	-	Ref.	-	Ref.	-
Tertile 2	0.189(0.040, 0.889)	**0.035**	0.153(0.032, 0.737)	**0.019**	0.171(0.035, 0.848)	**0.031**
Tertile 3	1.023(0.406, 2.578)	0.962	1.110(0.409, 3.012)	0.837	1.387(0.433,4.446)	0.582
*P_for trend_*		0.883		0.851		0.581
PROM						
DII (continuous)	1.042 (0.864, 1.256)	0.668	1.060 (0.872, 1.290)	0.558	1.028 (0.819, 1.291)	0.811
Tertile 1	Ref.	-	Ref.	-	Ref.	-
Tertile 2	1.378 (0.713, 2.661)	0.340	1.492 (0.753, 2.954)	0.251	1.409 (0.692, 2.870)	0.345
Tertile 3	1.089 (0.554, 2.140)	0.805	1.161 (0.571, 2.361)	0.681	1.053 (0.479, 2.314)	0.897
*P_for trend_*		0.838		0.692		0.939

DII, dietary inflammatory index; SGA, small-for-gestational-age; AGA, appropriate-for-gestational-age; LBW, Low birth weight; PROM, Premature rupture of membranes; Ref., reference. Crude model: Unadjusted. Crude model: Unadjusted. Adjusted model 1: Adjusted for age, pre-pregnancy BMI, primiparity status, and gestational weight gain. Adjusted model 2: Adjusted for total energy intake based on Model 1. P_for trend_ across the DII tertile is calculated using the median intake of each tertile as a continuous term. Bold values indicate that the p-value is less than 0.05, indicating statistical significance.

### 3.5 Sensitivity analyses of the association between DII and fetal distress in GDM pregnant women

Sensitivity analyses were performed to assess the robustness of the association between dietary inflammatory index and fetal distress. After additionally adjusting for nutritional counseling frequency and OGTT glucose levels (1-h and 2-h), the results remained materially unchanged (Tables 5 and Supplementary Table S3), and were consistent with the primary analyses.

**Table 5:** Logistic regression analysis of E-DII scores and the risk of fetal distress in GDM pregnant women.

E-DII tertiles	Crude model		Adjusted model 1		Adjusted model 2	
	OR (95% CI)	*P*	OR (95% CI)	*P*	OR (95% CI)	*P*
Fetal distress						
E-DII(continuous)	1.065(0.716, 1.583)	0.757	1.079(0.705, 1.652)	0.725	1.471(0.507, 4.270)	0.698
Tertile 1	Ref.	-	Ref.	-	Ref.	-
Tertile 2	0.092(0.012, 0.731)	**0.024**	0.092(0.011, 0.742)	**0.025**	0.078(0.009, 0.702)	**0.023**
Tertile 3	1.125(0.455, 2.784)	0.798	1.272(0.481, 3.364)	0.628	1.471(0.507, 4.270)	0.447
*P_for trend_*		0.690		0.592		0.517

E-DII, energy-adjusted dietary inflammatory index; Ref., reference. Crude model: Unadjusted. Adjusted model 1: Adjusted for age, pre-pregnancy BMI, primiparity status, and gestational weight gain. Adjusted model 2: Adjusted for nutritional counseling frequency, OGTT-1 h, and OGTT-2 h based on Model 1. P_for trend_ across the E-DII tertile is calculated using the median intake of each tertile as a continuous term. Bold values indicate that the *p*-value is less than 0.05, indicating statistical significance. E-DII tertiles were defined as T1 ≤ 0.929, T2 0.929–1.971, T3 > 1.971.

To account for total energy intake, the energy-adjusted dietary inflammatory index (E-DII) was used as an alternative exposure. No significant association was observed for the continuous E-DII in either the crude model (OR = 1.065, 95% CI: 0.716–1.583, *p* = 0.757) or the adjusted model (OR = 1.079, 95% CI: 0.705–1.652, *p* = 0.725). In tertile analyses, T2 was associated with a significantly lower risk compared with T1 in both the crude model (OR = 0.092, 95% CI: 0.012–0.731, *p* = 0.024) and the adjusted model (OR = 0.092, 95% CI: 0.011–0.742, *p* = 0.025), while no significant association was observed for T3 (Table 5).

Given the limited number of fetal distress events (n = 22), Firth’s penalized logistic regression was additionally applied for the fetal distress outcome to reduce small-sample bias. The adjusted model included the same covariates as the primary analysis (age, pre-pregnancy BMI, primiparity status, and gestational weight gain). Results were broadly consistent with the main analyses. For DII, a lower risk of fetal distress was observed in T2 compared with T1 in the adjusted model (OR = 0.186, 95% CI: 0.035–0.670, *p* = 0.009), whereas T3 and the continuous DII score were not significantly associated with fetal distress (Table 6). Similar findings were observed for E-DII, where T2 showed a significantly reduced risk in both the crude model (OR = 0.131, 95% CI: 0.014–0.575, *p* = 0.004) and the adjusted model (OR = 0.128, 95% CI: 0.014–0.572, *p* = 0.005), with no significant associations for T3 (Table 7).

**Table 6:** Firth’s penalized logistic regression analysis of DII and risk of fetal distress in GDM pregnant women.

DII tertiles	Crude model		Adjusted model 1	
	OR (95% CI)	*P*	OR (95% CI)	*P*
Fetal distress				
DII(continuous)	0.931(0.686, 1.253)	0.638	0.917(0.669, 1.248)	0.583
Tertile 1	Ref.	-	Ref.	-
Tertile 2	0.226(0.043, 0.806)	**0.020**	0.186(0.035, 0.670)	**0.009**
Tertile 3	1.022(0.409, 2.554)	0.961	1.056(0.402, 2.766)	0.911
*P_for trend_*		0.878		0.903

DII, dietary inflammatory index; Ref., reference. Crude model: Unadjusted. Adjusted model 1: Adjusted for age, pre-pregnancy BMI, primiparity status, and gestational weight gain. P_for trend_ across the DII tertile is calculated using the median intake of each tertile as a continuous term. Bold values indicate that the p-value is less than 0.05, indicating statistical significance. Firth’s penalized logistic regression was applied only for the fetal distress outcome because of the limited number of events.

**Table 7:** Firth’s penalized logistic regression analysis of E-DII and risk of fetal distress in GDM pregnant women.

E-DII tertiles	Crude model		Adjusted model 1	
	OR (95% CI)	*P*	OR (95% CI)	*P*
Fetal distress				
E-DII(continuous)	1.062 (0.717, 1.580)	0.763	1.072 (0.708, 1.633)	0.743
Tertile 1	Ref.	-	Ref.	-
Tertile 2	0.131(0.014, 0.575)	**0.004**	0.128(0.014, 0.572)	**0.005**
Tertile 3	1.120(0.459, 2.761)	0.802	1.246(0.484, 3.238)	0.647
*P_for trend_*		0.683		0.588

E-DII, energy-adjusted dietary inflammatory index; Ref., reference. Crude model: Unadjusted. Adjusted model 1: Adjusted for age, pre-pregnancy BMI, primiparity status, and gestational weight gain. P_for trend_ across the E-DII tertile is calculated using the median intake of each tertile as a continuous term. Bold values indicate that the *p*-value is less than 0.05, indicating statistical significance. Firth’s penalized logistic regression was applied only for the fetal distress outcome because of the limited number of events.

Influence diagnostics based on the fully adjusted logistic regression model for fetal distress were additionally performed. Although several observations exhibited relatively high leverage values, no single observation exerted a disproportionate influence on the association between DII tertiles and fetal distress, as indicated by Cook’s distance values remaining below the conventional threshold. Detailed diagnostic statistics are presented in Supplementary Table S2. Overall, the sensitivity analyses were broadly consistent with the primary findings, indicating that the association between dietary inflammatory potential and fetal distress was robust to energy adjustment and alternative modeling approaches for rare events.

## 4 Discussion

This prospective cohort study examined the association between third-trimester DII scores and perinatal outcomes among women with GDM. The study found that women in the intermediate DII category (T2) exhibited a reduced risk of fetal distress compared with those in the T1 group, whereas no statistically significant association was found in the highest DII group (T3). However, the associations were not consistent across categorical, continuous, and trend-based analyses. In addition, restricted cubic spline models indicated a possible deviation from linearity, although the overall association between DII and fetal distress was not statistically significant. Collectively, these findings indicate that the observed pattern should be interpreted cautiously and does not support a robust dose–response relationship.

Regarding the influence of DII on fetal distress, pro-inflammatory diets are generally associated with a higher risk. Qin et al. reported a higher incidence of fetal distress among pregnant women with elevated DII scores (40). Kyozuka et al. found that a pro-inflammatory diet significantly increased the risk of fetal umbilical artery pH < 7.10 at delivery, suggesting that a high-DII diet may directly affect fetal heart rate patterns by inducing intrauterine inflammation (41). These findings provide biological plausibility for a potential link between dietary inflammatory potential and fetal well-being. However, our study suggested a possible non-linear relationship between DII and fetal distress, with reduced risk only in the moderate DII group (T2). The potential inconsistency between analytical approaches in our study may suggest that the association between DII and fetal distress is not strictly linear and may be influenced by model specification and limited statistical power. Although spline analyses indicated a possible non-linear trend, this signal was not robust across statistical models and may reflect random variation due to the relatively small number of events. Therefore, this finding should be interpreted as exploratory and hypothesis-generating. From a biological perspective, several hypotheses may be proposed. Dietary inflammatory potential has been associated with gut microbiota composition and systemic inflammation, which may in turn influence metabolic regulation in GDM and placental function through inflammatory mediators and microbial metabolites (42–44). However, these mechanisms remain speculative in the context of our findings and cannot be confirmed by the present data. Moreover, previous evidence has suggested that associations between dietary inflammatory load and inflammatory biomarkers may be non-linear (45), although whether such patterns translate into clinically relevant perinatal outcomes remains uncertain. Importantly, the limited number of fetal distress cases in our cohort may have reduced the statistical power to detect stable associations and may contribute to the observed instability across models. Residual confounding and unmeasured dietary or socioeconomic factors may also have influenced the results. Therefore, these results should be regarded as preliminary evidence, and further large-scale prospective studies are required to clarify the relationship between dietary inflammatory potential and fetal distress in women with GDM.

With respect to birth-weight-related outcomes, including AGA, SGA, macrosomia, and low birth weight, our findings were largely consistent with previous research. de Andrade Miranda et al. (22) found no significant association between pro-inflammatory diets and the occurrence of SGA, a finding echoed by Casas et al. (46). Similarly, Killeen et al. (47) and Chen et al. (21) found no significant associations between E-DII and macrosomia. In contrast, some studies reported an increased risk of low birth weight in relation to higher dietary inflammatory potential. Notably, a cohort study with 307 pregnant women indicated that the pro-inflammatory diet group exhibited a 10.44-fold higher risk compared with the anti-inflammatory group (48), as well as a large study of 7,194 mother–infant pairs linking pro-inflammatory diets to low birth weight (49). The absence of such associations in our study may be attributed to the distinctive characteristics of our study population: all participants had GDM, a condition known to promote higher fetal growth trajectories, which may attenuate or mask the influence of diet-induced inflammation on fetal weight. Additionally, the modest sample size may have limited statistical power to detect associations between DII and low birth weight.

In addition, our study did not identify significant associations between DII and the risks of preterm birth or cesarean delivery. A secondary analysis of 434 mother–infant pairs from the Dublin-based Pregnancy Exercise and Nutrition Research Study likewise reported no relationship between E-DII and either mode of delivery or preterm birth (47), and a large European investigation spanning 24,861 mother–child dyads across seven cohorts similarly found no statistical association between DII and preterm birth (21). Collectively, these findings suggest that the influence of dietary inflammatory potential on preterm birth and cesarean delivery may be limited, or that detecting such relationships may require studies with substantially larger sample sizes. The etiology of premature rupture of membranes (PROM) is multifactorial, with inflammation playing a central mechanistic role (50, 51). Elevated inflammatory cytokine concentrations and broader inflammatory signatures have been consistently observed in the fetal membranes, amniotic fluid, and maternal circulation of individuals with PROM (52). However, no existing evidence has established a direct causal link between PROM and the DII, and our study likewise found no significant association between DII and PROM risk. Further targeted research is needed to elucidate the precise relationship and underlying mechanisms in this domain.

This prospective study focused on pregnant women with confirmed GDM, examining the dietary inflammatory status during late gestation among those who had already received dietary management, thereby filling a gap in evidence regarding DII in high-risk populations. Moreover, the study revealed a non-linear relationship between DII and fetal distress within the GDM cohort, offering new insights into the role of dietary inflammatory potential in pregnancy outcomes.

However, several limitations should be acknowledged. First, the DII was calculated based on 27 of 45 possible dietary parameters due to FFQ constraints, with certain components excluded because of low consumption frequency in Chinese pregnant women. Although this is unlikely to materially affect overall DII estimates, it may reduce measurement precision and limit comparability with studies using the full DII algorithm. Importantly, this study did not perform food group–based dietary pattern analyses; therefore, differences across DII tertiles cannot be directly interpreted in terms of specific dietary behaviors, which limits clinical interpretability. Second, dietary intake was self-reported using an FFQ; despite standardized one-to-one interviews, real-time clarification, and the use of visual aids, recall bias cannot be excluded. Third, the relatively small number of fetal distress events may have limited statistical power and increased the uncertainty of effect estimates; in addition, residual confounding from unmeasured variables cannot be ruled out. Although influence diagnostics did not identify any single highly influential observation, several observations with elevated leverage values were detected, warranting cautious interpretation. Finally, future studies with larger sample sizes and the incorporation of inflammatory biomarkers (e.g., CRP and IL-6) are needed to validate these findings and further explore potential non-linear associations, while also improving dietary assessment methods to enhance exposure accuracy.

## 5 Conclusion

This prospective cohort study examined third-trimester DII scores and perinatal outcomes in women with GDM. A non-linear association between DII and fetal distress was observed, with a lower risk in the intermediate tertile (T2) compared with T1 in categorical analyses, while no significant association was found for T3. No consistent associations were observed between DII and other pregnancy outcomes. Overall, DII demonstrated limited predictive value for major perinatal outcomes. However, its potential association with fetal distress warrants further investigation and may have potential implications for dietary management in women with GDM. Given the limited number of fetal distress events and the exploratory nature of this non-linear association, further validation in larger prospective studies is warranted.

## Data Availability

The raw data supporting the conclusions of this article will be made available by the authors, without undue reservation.
