# Supplementary material for: Association between the dietary inflammatory index and pregnancy outcomes in women with gestational diabetes mellitus: a prospective cohort study
Source: Front Nutr. 2026 Jun 26;13:1838318. doi: 10.3389/fnut.2026.1838318 (PMC13349769; doi:10.3389/fnut.2026.1838318)
Supplement: Supplementary file 1 [file Data_Sheet_1.ZIP › Data Sheet 1/Supplementary Materials/Supplementary Table S1-S3.docx]

**Table S1. Availability of the 45 DII Components in the Present Study**

| Food parameter | Available | Source |
| --- | --- | --- |
| Alcohol (g) | No | Not available |
| Vitamin B_12_ (μg) | Yes | SQ-FFQ |
| Vitamin B_6_ (mg) | Yes | SQ-FFQ |
| β-Carotene (μg) | Yes | SQ-FFQ |
| Caffeine (g) | No | Not available |
| Carbohydrate (g) | Yes | SQ-FFQ |
| Cholesterol (mg) | Yes | SQ-FFQ |
| Energy (kcal) | Yes | SQ-FFQ |
| Eugenol (mg) | No | Not available |
| Total fat (g) | Yes | SQ-FFQ |
| Fibre (g) | Yes | SQ-FFQ |
| Folic acid (μg) | Yes | SQ-FFQ |
| Garlic (g) | No | Not available |
| Ginger (g) | No | Not available |
| Fe (mg) | Yes | SQ-FFQ |
| Mg (mg) | Yes | SQ-FFQ |
| MUFA (g) | Yes | SQ-FFQ |
| Niacin (mg) | Yes | SQ-FFQ |
| n-3 Fatty acids (g) | Yes | SQ-FFQ |
| n-6 Fatty acids (g) | Yes | SQ-FFQ |
| Onion (g) | No | Not available |
| Protein (g) | Yes | SQ-FFQ |
| PUFA (g) | Yes | SQ-FFQ |
| Riboflavin (mg) | Yes | SQ-FFQ |
| Saffron (g) | No | Not available |
| Saturated fat (g) | Yes | SQ-FFQ |
| Se (μg) | Yes | SQ-FFQ |
| Thiamin (mg) | Yes | SQ-FFQ |
| Trans fat (g) | No | Not available |
| Turmeric (mg) | No | Not available |
| Vitamin A (RE) | Yes | SQ-FFQ |
| Vitamin C (mg) | Yes | SQ-FFQ |
| Vitamin D (μg) | Yes | SQ-FFQ |
| Vitamin E (mg) | Yes | SQ-FFQ |
| Zn (mg) | Yes | SQ-FFQ |
| Green/black tea (g) | No | Not available |
| Flavan-3-ol (mg) | No | Not available |
| Flavones (mg) | No | Not available |
| Flavonols (mg) | No | Not available |
| Flavonones (mg) | No | Not available |
| Anthocyanidins (mg) | No | Not available |
| Isoflavones (mg) | Yes | SQ-FFQ |
| Pepper (g) | No | Not available |
| Thyme/oregano (mg) | No | Not available |
| Rosemary (mg) | No | Not available |

**Table S2. Influence diagnostics for the association between DII tertiles and fetal distress**

| **Diagnostic statistic** | **Mean** | **Median** | **Maximum** | **Interpretation threshold** |
| --- | --- | --- | --- | --- |
| Cook’s distance | 0.028 | 0.001 | 0.746 | Values >1 may indicate highly influential observations |
| Leverage | 0.027 | 0.019 | 0.307 | Relatively high values may indicate influential observations |

Abbreviations: DII, Dietary Inflammatory Index.

Influence diagnostics were based on the fully adjusted logistic regression model for fetal distress. The model was adjusted for age, pre-pregnancy BMI, primiparity status, gestational weight gain, and total energy intake.

**Table S3 Logistic Regression Analysis of DII Scores and the Risk of Fetal Distress in GDM Pregnant Women**

| **DII tertiles** | Adjusted model | |
| --- | --- | --- |
|  | OR(95%*CI*) | *P* |
| **Fetal distress** | | |
| DII(continuous) | 0.968(0.647, 1.449) | 0.876 |
| Tertile 1 | Ref. | - |
| Tertile 2 | 0.112(0.020, 0.647) | **0.014** |
| Tertile 3 | 1.210(0.3334, 4.377) | 0.772 |
| *P_for trend_* |  | 0.660 |

Abbreviations:DII, dietary inflammatory index; Ref., reference.

Adjusted model: Adjusted for age, pre-pregnancy BMI, primiparity status, gestational weight gain, total energy intake, counseling frequency, OGTT-1h, and OGTT-2h.

*P_for trend_* across the DII tertile is calculated using the median intake of each tertile as a continuous term.

Bold values indicate that the p-value is less than 0.05, indicating statistical significance.
